# Bridging the gap between mechanistic biological models and machine learning surrogates

**DOI:** 10.1371/journal.pcbi.1010988

**Published:** 2023-04-20

**Authors:** Ioana M. Gherman, Zahraa S. Abdallah, Wei Pang, Thomas E. Gorochowski, Claire S. Grierson, Lucia Marucci

**Affiliations:** 1 Department of Engineering Mathematics, University of Bristol, Bristol, United Kingdom; 2 School of Mathematical and Computer Sciences, Heriot-Watt University, Edinburgh, United Kingdom; 3 School of Biological Sciences, University of Bristol, Bristol, United Kingdom; Goethe University Frankfurt: Goethe-Universitat Frankfurt am Main, GERMANY

## Abstract

Mechanistic models have been used for centuries to describe complex interconnected processes, including biological ones. As the scope of these models has widened, so have their computational demands. This complexity can limit their suitability when running many simulations or when real-time results are required. Surrogate machine learning (ML) models can be used to approximate the behaviour of complex mechanistic models, and once built, their computational demands are several orders of magnitude lower. This paper provides an overview of the relevant literature, both from an applicability and a theoretical perspective. For the latter, the paper focuses on the design and training of the underlying ML models. Application-wise, we show how ML surrogates have been used to approximate different mechanistic models. We present a perspective on how these approaches can be applied to models representing biological processes with potential industrial applications (e.g., metabolism and whole-cell modelling) and show why surrogate ML models may hold the key to making the simulation of complex biological systems possible using a typical desktop computer.

This is a *PLOS Computational Biology* Methods paper.

## Introduction

Mathematical mechanistic models have been used for centuries to understand and represent the natural laws that shape the world around us. Initially, the focus was on modelling specific phenomena and the mechanics underpinning them, but in time, these models became more complex and are now able to represent interconnected processes and the interaction with their environment, bringing us closer to building digital twins [1–3]. This complexity brings a number of challenges, among which their computational demand is one of the most pressing ones. Simulations of complex mechanistic models can take hours or days to run, making them unfeasible for real-time decision-making or sensitivity analysis. This can make users reluctant to utilise them, despite their predictive power.

**Fig 1:**
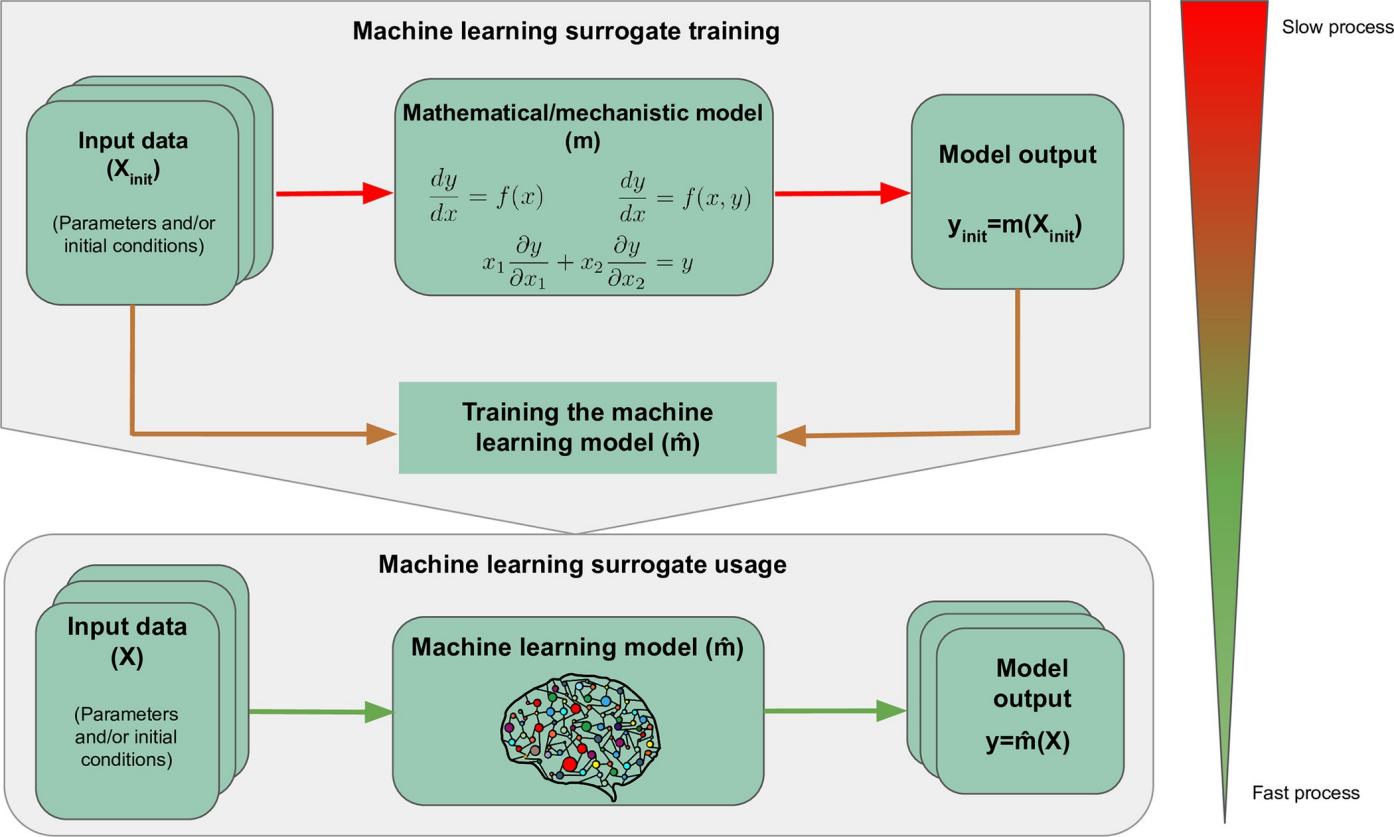
Schematic representation for training and using an ML-based surrogate model. The mechanistic model is simulated (the top process connected by red arrows) to obtain the input-output pairs that are used to train the ML surrogate. This training stage (the middle process connected by orange arrows) is an average process in terms of speed. Its complexity will depend on the ML algorithm used, the complexity of the data preprocessing steps, and the quantity of training iterations needed to obtain a satisfactory accuracy. Once this is achieved, the ML model can be used for all future predictions, effectively approximating the mechanistic model while running several orders of magnitude faster. The green arrows at the bottom of the figure represent this final (fast) process.

Here, we show how the high computational demand of some mechanistic models can be alleviated by using machine learning (ML) surrogates as a proxy. ML surrogates, also known as emulators or metamodels, are simpler models that approximate the behaviour of a mechanistic one. Usually, they take as input the initial conditions and/or parameters of the mechanistic model and they predict some or all of its outputs. Once the ML surrogate is trained and validated, it can replace the original mechanistic model in all future simulations, with the added advantage of making the simulations several orders of magnitude faster. The process of training and using an ML surrogate is shown in Fig 1. To create a surrogate, it is necessary to decide what output needs to be predicted and which inputs of the mechanistic model will be varied. Often it is not necessary to predict everything that the mechanistic model outputs, making the training process of the surrogate faster and more efficient. Once these choices are made, several simulations of the mechanistic model can be run by varying the chosen inputs to create input-output pairs for training, testing, and validating the ML model. The data is usually split such that 80% to 90% is used for training, and the remaining 10% to 20% is used for testing. To validate the surrogate model, one can either split the training data further such that a fixed percentage (usually 10% to 20%) is used only to validate the model [4–6] or implement cross-validation [7–11]. In terms of computational demand, the speed of the training phase relies on the ML model used and the number of iterations needed to obtain a satisfactory accuracy.

The improvement in computational speed that ML surrogate models achieve is particularly useful when predictions are needed in real-time [9,10,12] or when large numbers of simulations have to be run, for example, to explore a model’s parameter space [13–15]. It is important to acknowledge that simplified models can also lead to further improvements of the original ones. For example, different types of surrogate models have been used to analyse the uncertainty in the structure and the predictions of mechanistic models [16,17]. Also, while building the surrogate, it is possible to gain further understanding of the model’s relationship between inputs, parameters, and outputs, discovering for example, insensitive parameters [18].

Biological processes have been modelled mechanistically for decades and can be split based on 2 paradigms. The first one classifies them by scale [19], considering the cell as the “unit” for measurement, it is possible to create models at the subcellular, cellular, or macroscopic level. Subcellular models describe the evolution of individual physical and biochemical states of a cell [20]. Cellular-level models describe the interactions among different molecules and processes within cells, and macroscopic-level models describe processes that involve groups of cells [19]. The second paradigm classifies biological models based on the mathematical formalism that they use [21]. Biological processes are commonly modelled using ordinary differential equations (ODEs) [22–24], partial differential equations (PDEs) [25–27], agent-based modelling [21,28], cellular automata [29,30], stoichiometric matrices [31,32], stochastic techniques (for example, stochastic differential equations, SDEs) [23], or rule-based methods [33]. Details of each modelling approach are addressed in [19,21].

The main aim of this review is to bridge the gap between computationally demanding mechanistic models that describe biological systems at different cellular levels and the potential use of ML surrogates. First, we will review the performance of different ML-based surrogate models, while analysing their advantages and disadvantages when applied to ODE, SDE, and PDE-based mechanistic models. Then, we will discuss the benefits of using surrogate ML models in general, their limitations, and the future avenues for improving these models and making them more usable by scientists from different fields and communities. Finally, we will present how ML surrogate models can be relevant to approximate mechanistic models in the context of bioengineering industrial applications.

## ML as a surrogate in systems biology

ML-based surrogates were used to approximate mechanistic models of biological systems based on ODEs, SDEs [4,7], and PDEs [5,8–10,13]. These applications will be summarised below, focusing on the methodology and results of each study. Table 1 presents a summary of the surrogate ML models used for biological applications and their performance relative to the original mechanistic model they approximate. An overview of relevant methodological studies that apply ML-based surrogate modelling to mechanistic models from other engineering disciplines is presented in Table 2. The rest of this section will explain how these results were obtained.

**Table 1:** Summary of the performance and methodologies of the ML surrogates of the systems biology models.

Original model description	Surrogate algorithm	Surrogate accuracy	Improvement in computational time	Reference
SDE model of the MYC/E2F pathway [22,23]	LSTM	*R*^2^ 0.925–0.998	-	[4]
Heterotrimetric G-protein of budding yeast [24]	Orthogonal polynomial basis from the generalised polynomial chaos (gPC)	MAE 2.5 x 10^−2^	20% reduction in CPU time	[7]
Pattern formation in *E*. *coli* [25]	LSTM.	*R*^2^ 0.987–0.99	30,000 fold acceleration	[4]
Pheromone-induced cell polarisation in budding yeast [26]	Orthogonal polynomial basis from the generalised polynomial chaos	MAE 0.14	180-fold reduction	[7]
Statistical model for aorta shapes [27,34,35]	PCA, bidirectional neural network, feedforward neural network	Avg. MAE 0.533 mm	4 orders of magnitude	[8]
Risk for ascending aortic aneurysm [35]	PCA, bidirectional neural network, feedforward neural network	Avg. MAE: 1.366 KPa	4 orders of magnitude	[9]
Stress analysis of arterial walls under atherosclerosis [5]	Feedforward neural network	Test error 9.86%	-	[5]
Normal left ventricle [36–38]	XGBoost or Cubist	MAE for volume 1.495, MAE for pressure 1.544	2–3 orders of magnitude	[10]
Human left ventricle model [13,39,40]	Gaussian process	MSE 0.0001	3 orders of magnitude	[13]
Human left ventricle [11,39]	K-Nearest Neighbour, XGBoost, Multilayer Perceptron	*R*^2^ 0.999 (for the XGBoost and Multilayer Perceptron)	3–4 orders of magnitude	[41]
Physiology models: Small and HumMod [42]	SVM regression	Average error for Small: 0.05Â ± 2.47 and for HumMod: −0.3 +/- 3.94	6 orders of magnitude	[43]

The accuracy of the models is reported in terms of mean average error (MAE), mean squared error (MSE), coefficient of determination (*R*^2^), and average error.

**Table 2:** Summary of the performance and methodologies of the ML surrogate models that describe engineering processes with methodologies that can be extended to surrogates of biological models.

Original model description	Surrogate algorithm	Surrogate accuracy	Improvement in computational time	Reference
Simplified land model in the Energy Exascale Earth System Model [6]	Singular value decomposition and neural network	MSE loss: 0.02	3–4 orders of magnitude	[6]
Stochastic analysis of time-dependent PDEs solved using Monte Carlo method [44]	Convolutional autoencoder and feed forward neural network	MSE loss: 0.03	81 times	[44]

Accuracy is reported as mean squared error (MSE).

### ML surrogates of ODE and SDE systems

Dynamical systems that evolve only in 1 dimension are usually modelled using ODEs or SDEs. ML surrogates were successfully applied to approximate systems biology models based on both types of techniques. For example, Renardy and colleagues [7] built a surrogate based on an orthogonal polynomial basis from the generalised polynomial chaos (gPC) using the least square approximation for the heterotrimeric G-protein cycle of budding yeast. Once trained, the surrogate was used to compare its outputs to experimental data, with results showing high consistency between the 2, a mean absolute error (MAE) of 2.5*10^−2^, as well as a 20% reduction in CPU time. The authors noted that this speed-up in computational time might not be high enough to balance the time invested in building the surrogate, suggesting that it is important to approximate a priori the expected improvements in computational time that a surrogate might bring.

In [4], Wang and colleagues used a surrogate model based on a long short-term memory (LSTM) deep neural network to replicate the behaviour of an SDEs model describing the MYC transduction pathways with E2F regulator (MYC/E2F) in cell-cycle progression. The mechanistic model consisted of 10 SDEs and 24 trainable parameters. These parameters were varied using some prespecified ranges, and simulations were run to produce the training data necessary for the surrogate. The output to be predicted by the surrogate is a kernel distribution of the final values of each of the 10 variables. Apart from the high accuracy of the model and the improvement in computational time (Table 1), this analysis shows how surrogate ML models can be used to replicate stochastic systems. Specifically, different runs of the mechanistic model using the same parameters will produce different concentration levels for each molecule, but the distribution of these concentrations is deterministic for a sufficiently large number of runs. This suggests that each combination of parameters leads to a distribution for each molecule, corresponding to the spatial pattern output of the SDE model, which can be predicted by the surrogate neural network.

### ML surrogates of PDE systems

Complex dynamical systems that evolve in 2 or more dimensions are often modelled using PDEs. Traditionally, these models are solved numerically using finite element analysis (FEA) methods [45]. In this section, we will review the applicability of ML surrogates to mathematical models described by PDEs for molecular biology processes [4,7] and biomedical systems [5,8–10,13,41,43].

#### Applications in molecular biology

In molecular biology, surrogate models based on LSTM neural networks [4,46] were built to predict pattern formation in *E*.*coli* programmed by a synthetic gene circuit [25] represented as the spatial distribution of different molecules. The LSTM took as input the parameters of the mechanistic model and was trained to predict 2 outputs: the logarithm of the peak value of the profile of different molecules and their normalised profile. The authors reported a 30,000-fold computational acceleration [4], the LSTM being successfully used to identify new patterns by screening 10^8^ parameter sets in 12 days (compared to thousands of years which is how long it would have taken for the PDE model to achieve this). To improve the robustness of the ML model, the authors also proposed a reliability metric based on a voting system across different neural networks trained in parallel. This was an important addition to surrogate modelling that can prove particularly useful in cases when the surrogate is uncertain about a prediction, since the mechanistic model can be run instead.

In [7], Renardy and colleagues presented a technique based on polynomial surrogates using a Legendre polynomial basis that was applied to a spatial model of pheromone-induced cell polarisation of budding yeast. Once the polynomial surrogate was fit, it was used to compute parameter sensitivities and perform rapid Bayesian parameter inference. Using the surrogate, it was possible to run simulations that would take approximately 200 years to run using the mechanistic model. Furthermore, the surrogate facilitated the convergence for the distribution of 15 parameters in only a few hours using Bayesian inference.

#### Applications in organ modelling and physiology

Biomedical engineering is a field where surrogate models have been built extensively over the past decade, with a particular focus on biophysical models of the heart. Modelling myocardial properties that can help in making real-time clinical decisions could contribute to understanding and treating heart diseases [47]. Several mathematical models of the myocardium could be used for these aims. However, most are restricted by their high computational demand. Several studies suggest that these limitations can be addressed by implementing surrogate models based on different ML algorithms [5,8–10,14,15,41,48,49].

For example, in [8], Liang and colleagues built an ML surrogate of the FEA method to estimate the zero-pressure geometry of the human thoracic aorta. The input (i.e., a pair of shapes) and output were first encoded as a set of scalars using principal component analysis (PCA). Then, the nonlinear mapping between the encoded input and output was performed using a feed-forward fully connected neural network. Lastly, the output was decoded again using PCA. It was shown that ML surrogates could enable real-time applications of the model, with prediction time under 1 s and an average mean absolute error of 0.533 mm. A similar approach was used by Liang and colleagues [9], the main difference being that here the model takes 1 shape as input, while in [8] a pair of input shapes was used.

Two deep learning approaches were tested to build a surrogate that predicts the point-wise distribution of stress on the arterial walls under atherosclerosis in [5]. The inputs of the surrogate model were parameters describing the geometry and arterial pressure, and the outputs were point-wise stress distributions. The performance of a feed-forward neural network was compared against that of a convolutional neural network, with the first outperforming the second. Similarly to other studies [10,48], the authors performed a features’ importance analysis by adjusting 1 input feature at a time and studying the impact of these changes on the accuracy of the deep learning model. This approach revealed expected correlations between arterial pressure and stress, but also less obvious ones such as the fact that lipid pool information had more impact on maximum stress compared to calcium deposits. This suggests that besides their predictive power, ML surrogates can also unravel some dynamics of the system that have not been studied previously.

Cai and colleagues [41] also used simulations of the LV diastolic filling with the aim of estimating model parameters. Features were first projected into a lower dimensional space, and 3 different ML models (K-nearest neighbour, XGBoost, and a multilayer perceptron) were tested to assess how well they learn the pressure-volume and pressure-strain relationships. The computational cost of simulations was reduced by 3 to 4 orders of magnitude when using the ML surrogate. Davies and colleagues [13] used 2 interpolation methods and 2 loss functions to estimate the material properties of a healthy volunteer’s left ventricle using only non-invasive data. Minimising the loss between the biomechanical model’s output and the emulator produced an estimate of the unknown parameters. Two loss functions were used: the Euclidean loss function that assumes that the outputs are independent and a Mahalanobis distance-based loss function that allows for correlation across outputs. The best results were achieved using local Gaussian process interpolation and the Euclidean loss function. The reported mean square error (MSE) was 0.0001, and the computational time was reduced by approximately 3 orders of magnitude, from weeks to a quarter of an hour.

Another proof-of-concept for the usability of surrogate modelling assessed the applicability of ML models to emulate 2 physiology mathematical models, Small and HumMod [42,43]. Support vector machine (SVM) regression models were used to map the parameter samples to the drop in mean arterial pressure. The accuracy of these surrogates was calculated with respect to the drop in mean arterial pressure observed after running the original mathematical model. Further error analysis showed that there was no significant difference between the performance of the ML model and the mechanistic one. The authors also compared the time complexity of the 2 approaches and showed that the ML model could make predictions approximately 6 orders of magnitude faster than both dynamical models.

Besides the improvements in computational demand, the studies presented in this section also address other important modelling aspects such as building surrogates of stochastic models, implementing reliability metrics [4], performing parameter sensitivity, inference [7], and feature importance analysis [5]. Furthermore, since biological systems are often high dimensional, dimensionality reduction is another important modelling aspect that has been combined with surrogate-based ML models in studies describing both biological [8,9,41] and other engineering systems [6,44]. To help with deciding whether such analysis can bring value to a study and understand what are the options when deciding on the algorithms and techniques to be used, we will next review some technical aspects that can help to design optimal models.

## Building, training, and using ML surrogates

Each of the aforementioned studies has its own set of limitations. Some of these are domain-specific and rely heavily on the knowledge of domain experts. For example, some methods cannot be used in a clinical setting yet because they were only trained on myocardial models coming from a patient with specific characteristics, and hence, they do not include inter-patient variability. Other limitations and design matters are more general, being common across surrogate models regardless of their application area. These technical aspects addressing the design and training of ML surrogates are discussed below.

### Active learning

**Fig 2:**
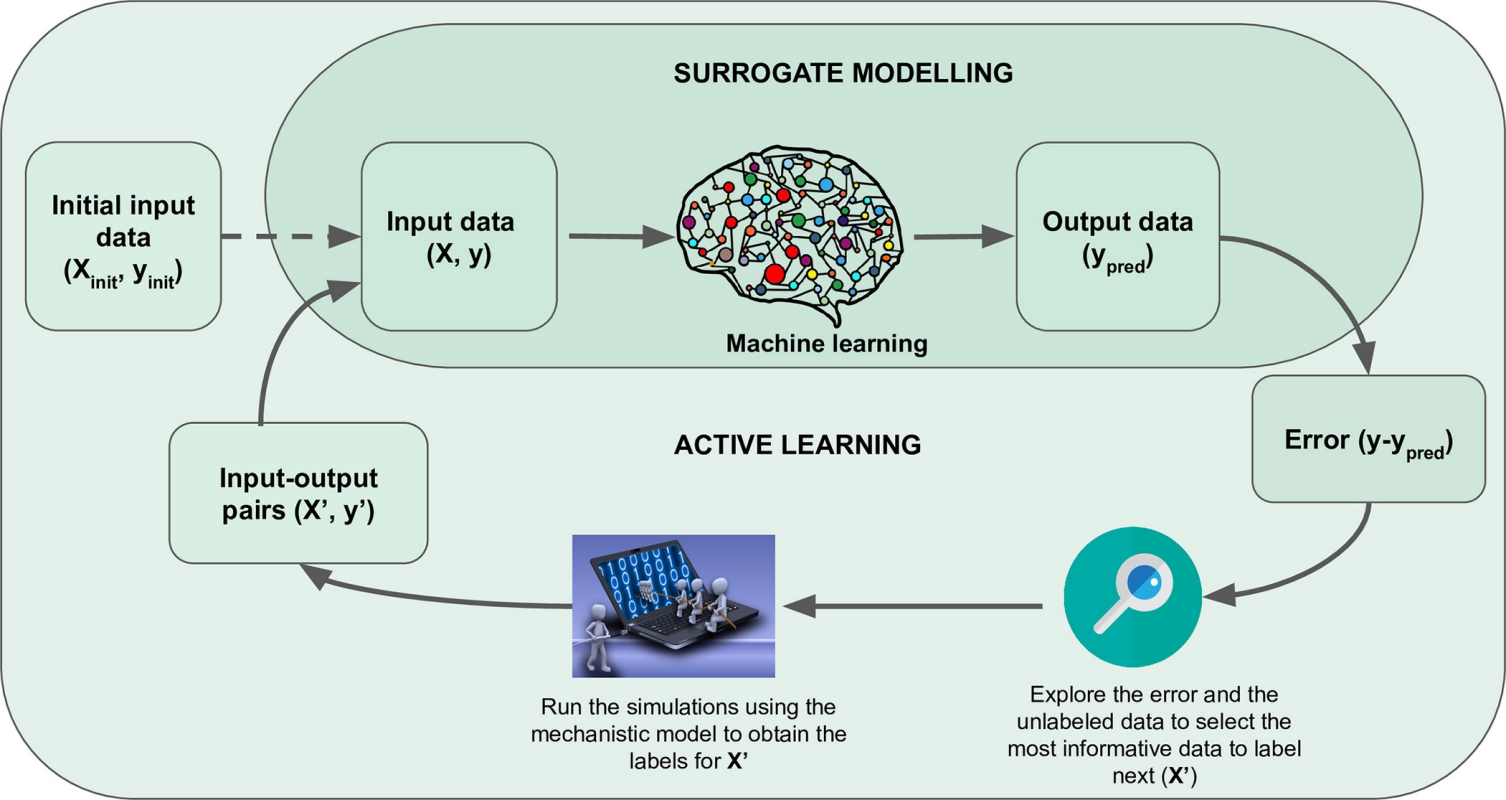
Schematic representation of how active learning and ML surrogates can work together. Initially, an ML model is trained on a set of data generated by some initial simulations of a mechanistic model (*X*_*init*_, *y*_*init*_), which are equivalent to (*X*, *y*) for this initial step. The ML model is used to make predictions (*y*_*pred*_). The estimated error between the prediction of the mechanistic model (*y*) and that of the ML model (*y*_*pred*_) is used to select a subset from all the possible input data that has not been used to make predictions using the mechanistic model in the past (X’). The mechanistic model is run using X’ as input to obtain a new set of input-output pairs (*X*, *y*), equivalent to the newly generated (*X*′, *y*′), that when included in the ML pipeline are expected to reduce the estimated error (*y*−*y*_*pred*_).

Given that surrogates are built to avoid running expensive simulations many times, it is important to minimise the number of simulations needed to train the ML model while keeping them as informative as possible. In active learning, a model can choose the data that it will learn from next, based on the information it gained from previous training examples. A summary of the process combining surrogate modelling and active learning is shown in Fig 2. Active learning has been applied together with surrogate models in engineering studies where the original mechanistic models were based on PDEs [50,51]. For example, in [50], Pestourie and colleagues built a neural network-based surrogate for the PDE model representing the Maxwell equations for composite materials and used an active learning algorithm that selects new training points from the parameter space where the estimated model error was higher. This error was recalculated after the training set was updated with new data obtained by running the simulations using the mechanistic model. The active learning approach was compared to a baseline where the training set was randomly sampled from the mechanistic model’s parameter space. The active learning surrogate matched the numerical integration result more closely, using 1 order of magnitude less training data compared to the surrogate trained on randomly sampled points.

Lye and colleagues [51] used deep learning surrogates and active learning to solve the constrained optimisation problem of 3 systems: optimal control problem for a nonlinear ODE, parameter identification for the heat equation, and shape optimisation of airfoils subject to the Euler equations. The algorithm presented is called iterative surrogate model optimization (ISMO), where a deep neural network queries a standard optimisation algorithm, quasi-Newton approximation, to provide training examples that will minimise its error. The ISMO algorithm outperformed the purely deep neural network surrogate in terms of error decay and robustness to parameter change and the standard optimisation algorithm for aerodynamic shape optimisation by more than an order of magnitude [51]. Other studies such as [52] presented strategies for building surrogate models with active learning using iterative parallel computations on single-core, multi-core, and multi-node architectures. These approaches can further speed up the modelling process.

### Designing the surrogate ML model

Choosing the right ML model to build the surrogate is an essential step in the process and it can be approached in several ways. First, the choice depends on what is the output to be predicted. In most of the use cases presented in Tables 1 and 2, the surrogate was used to predict a continuous output. This is the main reason why the corresponding ML algorithms are regression models. However, the studies reviewed above show that there is no consensus regarding the best regression algorithms to use. Tables 1 and 2 show that neural network methods perform very well, with an *R*^2^ of up to 0.99. However, they need a significant amount of data to be trained, meaning that more simulations of the mechanistic model need to be run, and are lacking explainability. On the other hand, decision tree-based methods have the advantage of being interpretable to some degree since they can output the features’ ranking, and perform similarly to the deep learning models in [41]. Algorithms such as Gaussian processes are both interpretable and can estimate the uncertainty in the predictions, which is why they are preferred in some cases.

If the output to be predicted by the surrogate is a discrete or categorical value, classification models are more appropriate. As in the case of regression, choosing the best algorithm depends on several factors such as the dimensionality and the amount of data available, whether uncertainty quantification and interpretability are important for the problem being addressed, and also how much technical ML knowledge the person building the surrogate has. These last 2 aspects are addressed in more detail in the sections regarding model usability and interpretability.

Since most of the mechanistic models presented here describe the dynamics of different systems, it is expected that an increasing number of future models will be based on time-series data. Such data often needs to be treated differently compared to tabular data. With the rise of computer power and deep learning techniques, there has been a lot of progress in the field of time-series analysis and prediction. Several reviews outline the state of the art when it comes to time-series forecasting algorithms [53–55] and time-series classification algorithms [56–58], some with the aim to make them interpretable [59–61].

One aspect that was mentioned in many of the studies presented in the previous section is related to the dimensionality of the data. When the input or output data is high dimensional, before training any ML model, it is good practice to apply some dimensionality reduction techniques [62,63] in order to address the curse of dimensionality issue [64] and overfitting. In fact, this was done in some of the studies presented in Tables 1 and 2 [6,8,9,41,44]. Another approach when dealing with high-dimensional data is to choose the inputs/outputs that the ML model will be trained on based on expert knowledge. This is less analytical, but in some cases, it can be more appropriate. When the output to be predicted by an ML model is multidimensional, it is also possible to build the model such that it predicts multiple outputs. The feasibility of this approach depends on the dimensionality of the mechanistic model. In [65], Xu and colleagues reviewed different methods and challenges regarding multi-output ML approaches, focusing on assessing the algorithms based on volume, velocity, variety, and veracity, all being important characteristics for models of biological and medical systems.

Another important aspect to be considered when it comes to the design of ML surrogates is the quantity of data used for training. Training data can be augmented by running more simulations (potentially using active learning to optimise this process), by using analytical data augmentation techniques, or when possible by adding matching experimental data to the synthetic data already used. To the best of our knowledge, the latter has not been done before. However, augmenting experimental data with synthetic one was done in [66], and in the training phase of their ML model, it had a positive impact on the performance. This data augmentation technique may not improve the accuracy of the surrogate when compared to the mechanistic model, but it may help the surrogate outperform the mechanistic model when tested against experimental observations, therefore, making the surrogate model more generalisable.

Different approaches may be considered when the mechanistic models to be emulated are not fully deterministic. Surrogate models have been used to approximate stochastic mechanistic models, and it was shown that if sufficient simulations are run, the distribution of the output of these models is approximately deterministic [4]. Another approach for building surrogates of stochastic models is to include the random seed that was used for the simulations as an input when training the ML model [67].

### Model usability

To train the ML surrogates, the user needs to be able to run the mechanistic model and train an ML model. Depending on the usability of the original model, its complexity and the user’s knowledge regarding ML, this could take more time than running the computationally expensive mechanistic model [7]. This suggests that it would be highly beneficial to include a reproducibility metric as part of surrogate modelling studies. For the initial training of the surrogate model to be as efficient as possible, the mechanistic models should have clear instructions on how to simulate them under different conditions. Furthermore, the training of the ML model should be accessible to non-experts. Once the training data is available, this is already possible using tools such as AutoML [68] or TPOT [69–71]. However, despite the ability of these tools to optimise for the ML model with the highest accuracy, they can limit the freedom of the user when it comes to designing the training process, for example, by making it challenging to control overfitting.

The other important aspect that needs to be addressed when we discuss usability and reproducibility is how easy it is to deploy the already-built surrogate, to re-train it, or slightly modify its scope. We believe that as surrogate modelling will be used more widely and becomes part of experimental or clinical pipelines, it is important to think about its tunability. Therefore, the code used to build the surrogates should be publicly available, well structured, and the ML pipeline presented clearly.

### Interpretability

Surrogate ML models can also help explain the behaviour of dynamical systems [5,10,48]. With the recent progress in the area of explainable artificial intelligence, once predictions are made, it becomes possible to interpret for example whether all input data impacts the prediction [72]. Furthermore, it is possible to understand which features influence the prediction the most and quantify this impact by investigating whether an increase in the value of one feature changes the prediction, and in the case of regression models whether the prediction is generally increased or decreased. Such methods could outline some behaviours of the system that were not previously known, especially when experimental data are used to train as well. In general, to make sure the results are robust, it can be helpful to apply different explainability methods and compare their results.

Above, we described the way surrogate machine learning models have been used in the literature and how the design and usability of such models can be enhanced. Using the information acquired from these sections, we propose future avenues for applying surrogate machine learning models to industrially relevant bioengineering.

## Further applications of ML-based surrogates in bioengineering

Given the potential of metabolic and whole-cell models in designing novel renewable biofuels [73,74] and drugs [75], as well as their versatility for minimal genome design [76,77], we further present our vision regarding the applicability of surrogate modelling for these types of mechanistic models.

Metabolism is among the most complex processes taking place in a cell. Genome-scale metabolic models include all the known information about the metabolism of an organism, such as genes, enzymes, reactions, and metabolites [78]. These models can be used not only to predict metabolic fluxes but also to understand genotype–phenotype interactions. In addition, they can have a significant impact on understanding strain development for the production of bio-based materials and chemicals, drug targeting, predictions of enzyme function, and modelling interactions among different cells [79]. Given the system-level complexity of these techniques, the models often end up containing thousands of genes, metabolites, and reactions that interact with each other. Metabolic kinetic models are even more computationally expensive since they predict the temporal behaviour of the process and they combine multi-omics data sets with reaction network models [80]. Some models require up to 7 h for 1 simulation, especially when a protein expression network is included [81]. This computational problem is amplified when complex organisms are modelled or several simulations have to be run.

**Fig 3:**
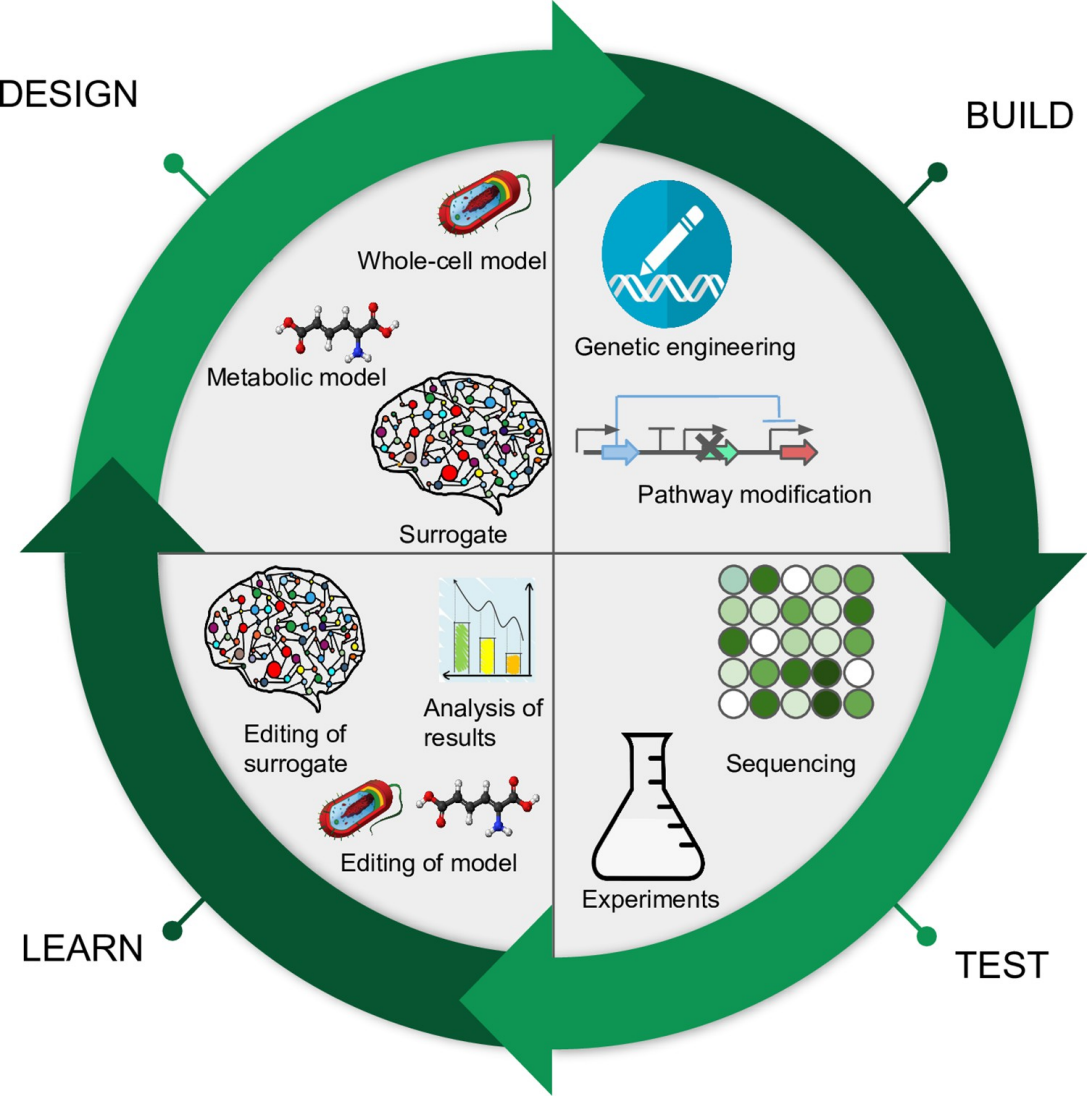
An example of the DBTL pipeline where the metabolic or whole-cell models can be replaced by surrogate models.

Often, metabolic models are used as part of a design–build–test–learn (DBTL) pipeline [82], corresponding to multiple combinations of inputs (Fig 3). This frequently involves a significant number of trial and error experiments, suggesting that ML surrogates of metabolic models would be particularly useful for such cases. For example, the ML surrogates can be trained on the initial state of the input variables of the mechanistic model and/or the parameters of the model, with the target variable being the desired phenotype to be predicted (a specific titer, rate, yield, or product). Once the training phase is completed, the surrogate can be used to approximate the original metabolic model. One of the challenges that may occur when implementing this framework is caused by the high dimensionality of metabolic models. This suggests that if the initial conditions are used as input for the ML model, it will be necessary to run several simulations to cover most of the variables’ space. According to [83,84], at least 4 to 5 times as many simulations as the number of variables are needed to avoid the curse of dimensionality. In the cases when this is possible, it can also lead to the discovery of interesting dynamics of the system since an explainable ML model can show which parameters and variables are influencing the phenotype the most. However, in other cases, running a high number of mechanistic simulations might defeat the purpose of building a surrogate model. For such situations, it is possible to reduce the dimension of the input by applying different dimensionality reduction techniques [85] or by manually selecting only the variables that are known to influence the desired phenotype [12].

Whole-cell models are mathematical models that include and link all the well-annotated genes and processes of a cell. Two such models have been published to date, for *Mycoplasma genitalium* [2] and for *Escherichia coli* [3,86], and more are underway [87]. The completeness of these models makes them particularly powerful since, when used in a DBTL pipeline, they facilitate the study and design of interactions among different cellular processes, something that a metabolic model alone cannot achieve. Similarly to metabolic models, whole-cell models have already been used for in silico minimal genome design [77], and we anticipate that they will change the paradigm for metabolic engineering and development of microbial chassis [88]. These models add some extra levels of complexity to the genome-scale metabolic models and therefore are even more computationally expensive [89], with a simulation time of 15 min to 24 h per cell (on a desktop computer). This makes applications that involve multiple cells growing over multiple generations prohibitively expensive due to their high computational time.

ML surrogate models can represent a strategy to address this challenge. For example, the input to the ML model can be defined as a subset of the initial conditions and parameters of the model, and the output as the phenotype to be predicted. This can be a continuous variable such as the growth rate of a cell, the production, titer or yield of different metabolites, or binary indicating, for example, whether a cell divides or not. Similarly to metabolic models, whole-cell models can have thousands of candidate variables that can be used as input to the ML model. As mentioned before, these can be reduced using dimensionality reduction techniques [85] or prior knowledge about the process under investigation [12].

## Conclusion

There is a growing number of studies in the literature showing how ML surrogates can be used to emulate mechanistic models of biological processes, both at the molecular and macroscopic levels. These show that, besides the performance of the surrogate models in terms of accuracy compared to numerical integration, and improvement in computational speed, it is also beneficial to consider other design aspects. First, it is important to assess whether the mechanistic model is complex enough to invest the time in building a surrogate [7]. The design of the protocol for obtaining the training data of the ML surrogate should consider aspects such as stochasticity and whether active learning could bring any value [4,50,51,67]. Furthermore, dimensionality reduction of the inputs and/or outputs of the ML surrogate [6,8,9,41,44] and parameter sensitivity analysis [7] not only can help to optimise the performance of the model, but also to unravel some information about the dynamics of the system.
